# New Landmarks to Slow the Progression of Chronic Kidney Disease

**DOI:** 10.3390/jcm12010002

**Published:** 2022-12-20

**Authors:** Marco Simonini, Giuseppe Vezzoli

**Affiliations:** 1 Nephrology and Dialysis Unit, IRCCS San Raffaele Scientific Institute, 20132 Milan, Italy; 2 Department of Nephrology and Dialysis, Vita Salute San Raffaele University, 20132 Milan, Italy

Chronic kidney disease (CKD) is a serious condition whose incidence is steadily rising, particularly in the Western world, due to the increasing prevalence of diabetes, hypertension, and obesity, which are nowadays the major causes of CKD in the Western population, as well as the aging of the population [1]. Currently, 800 million persons might have CKD, representing a prevalence of 11–13% of the global population [2].

Currently, regardless of the underlying etiology, CKD is a slowly progressive disease and leads to irreversible loss of nephrons, with a consequent progressive silent deterioration of renal function to end-stage kidney disease (ESKD) [3,4]. This is also associated with increased cardiovascular (CV) risk and premature death [5,6]. In addition to CV disease, which is the main cause of mortality and morbidity in CKD patients, osteoporosis and vertebral fractures may predict mortality risk in patients with stage 3–5 CKD [7,8].

Initial kidney injury can present in a wide range of severity, from asymptomatic hematuria to more complex and severe clinical pictures [9]. These different manifestations are, at least in part, due to the way the kidney reacts to the injury. Moreover, even if the initial disorder is inactive or promptly cured, progressive kidney disease may develop due to intrinsic hemodynamic factors and other mechanisms such as chronic inflammation, reduced regenerative capacity of the kidney, and fibrosis. Indeed, the kidney can adapt to its damage by increasing the single nephron filtration rate (sGFR) in the remaining normal tissue: this leads to a process known as “adaptive hyperfiltration” [10]. The adaptive hyperfiltration, although initially beneficial in terms of total renal function, appears to cause long-term damage to the glomeruli of the remaining nephrons, manifested mainly by proteinuria and progressive renal failure [11]. This mechanism is particularly important when renal function is less than 60 mL/min/m^2^ and appears to be a main determinant of ESKD in individuals whose original disease is inactive or resolved [12].

The decline in kidney function in patients with CKD is initially asymptomatic. However, with the progression of renal damage and the concomitant decline in renal function, various signs and symptoms may be observed, including volume overload, hyperkalemia, metabolic acidosis, hypertension, anemia, alteration of calcium–phosphorus equilibrium, and mineral bone disorders (CKD-MBD) [8,9]. In more advanced stages, usually with the onset of ESKD with a GFR < 15 mL/min/m^2^, symptoms and signs related to uremia appear, such as gastrointestinal manifestations (anorexia, sarcophobia, nausea and vomiting), systemic complication (pericarditis, neuropathy), and central nervous system abnormalities (loss of concentration, lethargy, seizures, and coma [10,13]).

The rate of CKD progression depends on the underlying disease, presence of comorbidities, treatments, socioeconomic status, genetic background, ethnicity, and other factors [14].

Currently, the identification of patients at risk for progression to CKD/ESKD and of the rate of worsening GFR is achieved through clinical monitoring and risk stratification, such as the CGA system (Cause of CKD, GFR and Albuminuria category) proposed in 2012 by the KDIGO guidelines [15]. However, the CGA classification system, although useful for quantifying the risk of specific CKD outcomes, has not yet been fully validated and applied in daily clinical practice and research studies. Moreover, this approach mostly remains for a long-term prognostic type, rather than a real marker of the progression of renal damage in the short term. Therefore, new algorithms have been proposed to detect CKD patients in the general population and identify those at risk of developing ESKD. An algorithm aimed at estimating the frequency of CKD in an Italian population was recently validated. It was built from an administrative database and was used to estimate the prevalence of CKD in the general population. An evolution of this or other algorithms focused on the clinical history of CKD patients could provide information about the evolution of these patients to ESKD [16]. Methods of artificial intelligence could contribute to improvements in the predictive power of these algorithms and identify new biological or social markers of evolutive kidney disease [17].

Other methods to investigate kidney function were made available by sonography that may estimate intrarenal and systemic atherosclerotic vascular dysfunction through the measurement of the intrarenal resistive index. After its first evaluation in proteinuric and atherosclerotic patients [18], recent findings confirmed that the intrarenal resistive index may be associated with kidney function decline in non-proteinuric CKD patients [19]. 

Indeed, it is now clear that to prevent the progression of CKD into ESKD, it is not enough to identify the etiology of the underlying kidney disease or to control the well-known risk factors, but the identification of new prognostic factors that can predict progression and lead to a more specific and early therapy is essential. Moreover, it has to be emphasized that, nowadays, no current therapy can prevent AKI or the AKI-to-CKD transition. Also in this case, a maladaptive repair after AKI is strongly associated with the development of CKD and long-term consequences. The prompt identification of patients at higher risk for late CKD progression and the development of new therapeutic interventions remain critical research goals.

Present-day therapy also has many shortcomings. Current treatments are mostly based on general nutritional interventions and lifestyle modification, blood pressure control, blood sugar control, and reduction in albuminuria. Although fully agreeable and of critical importance in controlling associated CV risk, current treatments may only delay disease progression, underscoring the need to develop new therapeutic approaches to stop or reverse CKD progression. Adopting measures to help prevent this process may slow down the disease’s evolution and even preserve long-term renal function. If these modalities are truly effective, their benefits are greater the earlier such therapy may start.

The current mainstay of CKD therapy is renin–angiotensin system (RAS) blockade using angiotensin-converting enzyme (ACE) inhibitors and/or angiotensin receptor blockers (ARBs) and, recently, sodium-glucose transporter 2 (SGLT2) inhibitors have been introduced. However, RAS blockade is indicated only in proteinuric or hypertensive nephropathies and delays, but does not prevent, CKD progression [20]. SGLT2 inhibitors have been shown to prevent CKD progression in patients with diabetes at high cardiovascular risk [21,22], and recent findings indicate that they may also exert this effect in non-diabetic CKD [23]. Finally, SGLT2 inhibitors and endothelin 1 receptor antagonists seem to reduce kidney fibrosis in animal models, but whether this is also true in humans has not been proven yet [24,25]. Analogously, mineralocorticoid receptor antagonist, finerenone, was found to delay CKD progression in diabetic patients probably through a decrease in renal inflammation and fibrosis processes [26].

In addition to pharmacological treatment, the low-protein diet is now considered as an integral part of therapy in CKD patients at pre-dialysis stages as it is effective in delaying ESKD and improving metabolic control and quality of life [27]. Management of a low-protein diet can now be modulated according to the necessities and preferences of patients and may even be adapted to vegan or vegetarian individuals using supplements with ketoacids. However, a dietary approach should be implemented because it is not universally prescribed by nephrologists. Patient nutritional status is another parameter not universally tested, that should be routinely evaluated in CKD patients to prevent the development of protein–energy wasting, consequent to an insufficient calorie and protein intake not balancing the anabolic condition of CKD patients [28].

In our opinion, starting from all this evidence, it should be clear that one of the main challenges that the nephrologist will face in the near future will be the early detection of markers of progression of kidney damage and the development of increasingly targeted therapies able to stop such progression or even allowing the kidney to regenerate.

In this regard, developments in knowledge about the ability of SGLT2 inhibitors to prevent or delay kidney fibrosis and cardiac damage will certainly be of primary interest. Moreover, clinical use of non-steroidal mineralocorticoid receptor antagonists should open a new field to reduce the risk of kidney function decline, kidney failure, cardiovascular death, non-fatal heart attacks, and hospitalization for heart failure in adults with CKD [29,30]. Finally, personalization of therapy and the use of non-pharmacological therapies, such as personalized nutritional approach [27,28,31] or the understanding of the role of microbiota [32,33], could also open up interesting new implications on the containment of GFR loss.

In addition to therapy, there is certainly the necessity, as mentioned above, for earlier and more accurate identification of patients at high risk of CKD progression. From this premise, new technologies that allow a better study of real renal residual “vitality” (such as more sophisticated imaging) [19] and identification of new biomarkers [34,35,36] that could be integrated into the current score for the progression could represent a breakthrough in the treatment of kidney disease. Moreover, new technologies, in particular artificial intelligence (IA) and machine learning, could really be a leap forward in properly stratifying patients according to individual risk [16]. The potential of these new technologies is not fully understood yet, and their impact could radically change our approach to certain clinical problems.

The challenge for the future in the treatment of CKD lies in our ability to understand more deeply its underlying pathophysiological mechanisms, to identify patients at risk early, and to propose increasingly effective and personalized therapies to interrupt that “slippery slope” that, in these days, inevitably leads from CKD to ERKD. 
